# Supplementary material for: ApoE e2 and aging-related outcomes in 379,000 UK Biobank participants
Source: Aging (Albany NY). 2020 Jun 8;12(12):12222–33. doi: 10.18632/aging.103405 (PMC7343499; doi:10.18632/aging.103405)
Supplement: Supplementary Table 3 [file aging-12-103405-s003..pdf]

SUPPLEMENTARY TABLE

Supplementary Table 3. ICD-10 Disease Codes.

Disease	ICD-10 codes	Notes
Age-Related Macular Degeneration	H353	
Anemia	D50-D53	
Atrial Fibrillation	I48	
Bladder Cancer	C67	
Breast Cancer	C50	
Colorectal Cancer	C18-20	
COPD	J42-J44	
Delirium	F05	
Dementia	F00; F01; F02; F03; G30	
Heart Failure	I50; J81	
Hypertension	I10-I15	
Hypothyroidism	E03	
Liver Disease	K70-K77	Any
Lung Cancer	C34	
Melanoma Cancer	C43	Malignant Melanoma
Coronary Artery Disease	I20–I25	MI or Angina
Osteoarthritis	M15.0; M15.1; M15.2; M15.9; M16.0; M16.1; M17.0; M17.1; M18.0; M18.1; M19.0	
Osteoporosis	M80; M81; M81.1; M81.2; M81.3; M81.4; M81.5; M81.6; M81.8; M81.9	
Parkinson's Disease	G20; F02.3	
Peripheral Artery Disease	I70.2; I70.9; I73; I74.2; I74.3; I74.4; I74.5; I79.2	Peripheral Vascular Disease
Pneumonia	J13; J14; J15; J16; J17; J18	
Prostate Cancer	C61	
Renal Failure	N18; N18.0; N18.3; N18.4; N18.5; N18.8; N18.9	
Rheumatoid Arthritis	M05; M06	
Stoke	G45-G46; I61; I63	Stroke/TIA
Type I Diabetes	E10	
Type II Diabetes	E11	
